# A Brief Report: Community Supportiveness May Facilitate Participation of Children With Autism Spectrum Disorder in Their Community and Reduce Feelings of Isolation in Their Caregivers

**DOI:** 10.3389/fpsyg.2020.583483

**Published:** 2020-11-10

**Authors:** Bethany D. Devenish, Carmel Sivaratnam, Ebony Lindor, Nicole Papadopoulos, Rujuta Wilson, Jane McGillivray, Nicole J. Rinehart

**Affiliations:** ^1^Deakin Child Study Centre, School of Psychology, Deakin University, Burwood, VIC, Australia; ^2^UCLA Division of Pediatric Neurology, David Geffen School of Medicine, UCLA Semel Institute of Neuroscience and Human Behavior, Los Angeles, CA, United States

**Keywords:** autism spectral disorder, community participation, children, caregivers, stress

## Abstract

Children with autism spectrum disorder (ASD) participate at lower rates in their community, and their caregivers experience higher levels of stress, in comparison to families of typically developing (TD) children. The social model of disability positions the environment as the central issue when children with disabilities are unable to participate, yet little is known about the relationship between poor community support, reduced community participation in children with ASD, and caregiver stress. This study examined caregiver perceptions of community supportiveness for the community participation of 48 children with ASD (aged 5–12 years), alongside caregiver-reported child ASD symptom severity, adaptive functioning, and caregiver stress. Community supportiveness predicted child involvement, but not attendance, when child characteristics were held constant. Caregiver perceptions of low community supportiveness significantly predicted caregiver feelings of isolation. The importance of modifying community programs to better support inclusion of children with ASD is discussed.

## Introduction

Children with autism spectrum disorder (ASD) often miss out on participating in activities such as community events, organized physical activity, informal interactions with other children, overnight visits or trips, and dance classes (Egilson et al., 2017; May et al., 2019), and their caregivers experience higher levels of stress than caregivers of typically developing (TD) peers (Hayes and Watson, 2013; Keenan et al., 2016). This reduced participation is a concern given community participation has been identified as a universal right for all children (UN General Assembly, 2007) and an important component of a child’s social, physical, and psychological development (Eime et al., 2013; Howells et al., 2019, 2020; May et al., 2019).

Children with ASD experience many barriers to community participation as a function of their everyday challenges with social interactions and communication, under or over reaction to sensory input, a strong desire for predictability and routine, and repetitive patterns of behavior (American Psychiatric Association, 2013). A range of studies have identified links between increased ASD symptom severity and low community participation (Bar-Shalita et al., 2008; Hochhauser and Engel-Yeger, 2010; Shattuck et al., 2011; Thompson and Emira, 2011; Krieger et al., 2018; May et al., 2018). These include isolation or peripheral involvement due to challenges with peer social interactions (Shattuck et al., 2011; Krieger et al., 2018), and decreased attendance due to sensory impairments (Bar-Shalita, et al., 2008; Hochhauser and Engel-Yeger, 2010; Krieger et al., 2018; May et al., 2018), anxiety (May et al., 2018), and repetitive and restricted behaviors and interests (Thompson and Emira, 2011; May et al., 2018). Adaptive functioning describes personal and social skills that support an individual’s ability to perform day-to-day activities independently (Sparrow et al., 2016) and encompass communication skills, daily living skills (linked to decreased community participation; Poon, 2011), socialization skills, motor skills (linked to decreased participation in community sports and leisure activities; Obrusnikova and Cavalier, 2011; May et al., 2018), and maladaptive behaviors (i.e., internalizing or externalizing behaviors) in reference to the ways in which greater difficulties with these skills disrupt day-to-day independence (Sparrow et al., 2016).

It has been argued from the perspective of the social model of disability that the environment plays a central role in determining whether children with disabilities and developmental challenges can participate fully (Shakespeare and Watson, 2002). As opposed to the medical model of disability, in which people are considered disabled as a function of their impairments, the social model shifts this focus to disability as a function of the barriers that prevents all people from being able to fully participate (Oliver, 2013). Consequently, proponents of this model are more interested in determining the ways in which environmental factors may inhibit or support participation than in identifying child “deficits” that may contribute to this inhibition. In keeping with this premise, research has explored community factors that inhibit the participation of children with ASD in their community. For example, adolescents with ASD who perceive the environment as low in safety and predictability have been found less likely to participate in their community (Krieger et al., 2018), and this is likely more pronounced in adolescents with ASD who have a higher need for adherence to routines and sameness (a symptom of ASD; American Psychiatric Association, 2013). Further, youths with ASD have reported that unclear implicit social demands act as a deterrent to their participation in community settings, as they feel intimidated when they are unsure how to understand, interpret, or react to social demands (Krieger et al., 2018), highlighting the likelihood that greater impairment in social skills will be linked to poorer participation in this population. Children with ASD can experience varying degrees of cognitive and communication challenges, and caregivers have reported that participation can be inhibited by the cognitive demands of community activities (Egilson et al., 2017). A number of other external factors that relate to both ASD symptom severity and adaptive functioning have been identified as barriers to the participation of children with ASD in their community, including lowered availability and suitability of appropriately trained community services and staff (Krieger et al., 2018).

Most previous research has not distinguished between child community participation measured as “attendance” and child community participation measured as “involvement.” Attendance captures a child’s presence within a program, but does not measure the depth of the participation, that is, how engaged and included they are (Imms et al., 2016). This is an important distinction, particularly when considering that ASD is characterized by challenges with initiating and responding to social interactions (American Psychiatric Association, 2013), in that they may attend regularly, yet not demonstrate the same level of involvement as their TD peers. Further, while research has identified ways in which symptom severity and adaptive behavior appear associated with their participation in community activities, there is an absence of research that examines both child characteristics and caregiver perceptions of community supportiveness in relation to both child attendance and involvement within a single sample. Examining both of these constructs together is important, as it is possible that mere attendance of a program may not improve social, physical, and psychological outcomes if involvement in the program is low. Further, understanding how the community environment supports or inhibits child participation is important for moving beyond a child deficit viewpoint to the social model of disability, as many of these factors may be amenable to modification. Participation in community activities such as organized and unstructured physical activity declines further in children with ASD as they move into adolescence (Simpson et al., 2019), highlighting the importance of early intervention in these settings to promote continued access to future participation opportunities. Community programs, such as organized physical activity, have been identified as promising psychosocial interventions for children with ASD (Rinehart et al., 2018) and identifying community barriers to participation may help inform modifications to community programs to support the full inclusion and participation of children with ASD, thereby improving social, physical, and psychological outcomes for children with ASD.

Understanding the mechanisms that may contribute to the relationship between caregiver stress and community participation is particularly important when taking into consideration the high rates of stress caregivers of children with ASD experience in comparison to caregivers of TD children (Hayes and Watson, 2013; Keenan et al., 2016). Caregivers of children with ASD tend to have fewer opportunities to engage in social interactions (Lecavalier et al., 2006; Myers et al., 2009) and face challenges in accessing community-based social supports (Sanders and Morgan, 1997). Further, caregivers who perceive social support received by themselves or their child as inadequate are more likely to experience high levels of stress (Gray and Holden, 1992; Sanders and Morgan, 1997; Siklos and Kerns, 2006; Siman-Tov and Kaniel, 2011). Indeed, research has found that a lack of social support, including stigmatization of a child’s behaviors or characteristics, can result in caregivers of children with ASD withdrawing from social situations (Sanders and Morgan, 1997; Eaton et al., 2016), thereby experiencing increased stress (Sanders and Morgan, 1997). Similarly, qualitative research has highlighted a lack of community support for the inclusion of children with disability as being one of the key challenges to caregiver well-being, with caregivers reporting experiencing high levels of stress and isolation, including feeling “labeled” by other parents, due to having to play the perpetual role of advocate for their child’s inclusion across multiple settings (Resch et al., 2010). Previous research found that caregivers rate community social organizations as offering the least helpful support (Hall and Graff, 2011), however, while a large body of research has examined the relationship between caregiver stress and social support from family and friends, to our knowledge, there is no quantitative literature that specifically examines the relationship between community supportiveness and stress in caregivers of children with ASD. In the closest study identified, child ASD symptom severity and community supportiveness could account for 16% of the variance in family coping scores (Hall, 2012).

To our knowledge, however, no previous research has examined whether lower levels of community supportiveness is linked to reduced community participation in children with ASD while holding ASD symptom severity and adaptive behaviors constant. Further, little research has examined these relationships in relation to the dual constructs of participation – attendance and involvement. Similarly, while previous qualitative research has identified possible links between lower levels of community supportiveness and higher levels of caregiver stress, this has not been evaluated while controlling for variability in ASD symptom severity and adaptive behaviors.

The aims of the current study are two-fold:

1. To examine the relationship between caregiver perceptions of community supportiveness and child participation (attendance and involvement);
2. To examine the relationship between caregiver perceptions of community supportiveness and caregiver stress.

Based on research findings children or youth with ASD, and their caregivers, identify a range of external barriers (i.e., cognitive and social demands of activities) to community participation (Egilson et al., 2017; Krieger et al., 2018), it is predicted that higher supportiveness of the community environment will predict higher levels of child community participation and involvement when holding child characteristics constant. Further, based on research identifying links between social support and caregiver stress (Gray and Holden, 1992; Sanders and Morgan, 1997; Siklos and Kerns, 2006; Siman-Tov and Kaniel, 2011) and community support and family coping (Hall, 2012), it is hypothesized that reduced community supportiveness and the higher symptom severity and adaptive behaviors that are often associated with community participation will be predictive of higher levels of caregiver stress.

## Materials and Methods

### Participants

The participants were 56 children aged 5–12 years and diagnosed with ASD, who were recruited as part of a larger pilot study examining the outcomes of participation in a community football program in metropolitan and regional Melbourne, Australia, and their caregivers. The present sample comprised of baseline data from children diagnosed with ASD who participated in the evaluation, half of whom were recruited from community football programs, and half who did not participate in organized physical activity but participated in their regular community activities. Participants were recruited through the community football participant database, research registries held by state peak disability bodies, private pediatric clinics, primary schools and special development schools, and social media. To be included in this study, children needed to be aged 5–12 years and have a pre-existing formal diagnosis of ASD. To receive a formal diagnosis of ASD in Victoria, Australia, a child must satisfy diagnostic and statistical manual of mental disorders (DSM) criteria, in which they have undergone assessment by a multidisciplinary panel and have their diagnosis confirmed by a pediatrician or child psychiatrist. Diagnosis was confirmed by caregivers during screening and diagnostic reports were sighted by researchers, where made available by caregivers. Baseline data from the broader study were utilized for the current study.

### Measures

Caregivers of participants completed a battery of questionnaires at baseline. Demographic data, including age, gender, and Full-Scale Intelligence Quotient (FSIQ) from age-appropriate Wechsler tests of Intelligence (e.g., Wechsler, 2011, 2012, 2014), were collected. Only the measures that are relevant to this study are reported below.

Participation Environment Measure Children and Youth (PEM-CY; Coster et al., 2011). The PEM-CY community average frequency and average involvement subscales were administered to measure child participation in the community, and the community average perceived environmental barriers and support subscale was administered to measure caregiver perceptions of community-level supports and barriers to their child’s participation. The average frequency and involvement subscales consist of 10 items related to activities typically performed in the community, specifically, community events, organized physical activity, unstructured physical activity, classes or lessons outside of school, organizations, clubs, groups or leadership activities, religious activities, “getting together” with other children in the community, and staying overnight (i.e., for a sleepover, holiday or camp). For average frequency, caregivers are asked to indicate how often their child participates on an 8-point scale, with responses including daily, few times a week, once a week, few times a month, once a month, few times in last 4 months, once in last 4 months, or never. For average involvement, caregivers are asked to indicate how involved their child is when participating in these activities on a 5-point scale, with responses ranging from minimally involved to very involved. The community average perceived environmental barriers and support subscale include nine items identifying a number of potential supports and barriers to participation, such as peer relationships, weather conditions and physical layout, and a further seven items identifying community resources. For the first nine items, caregivers were asked to indicate whether the environmental barriers and support made participation easier or harder for their child on a 4-point scale, with possible responses including “not an issue,” “usually helps,” “sometimes helps/sometimes makes harder,” and “usually makes harder.” Three of the resource items were asked to caregivers to indicate whether community resources were available and adequate on a 4-point scale, with possible responses including “not needed,” “usually, yes,” “sometimes yes/sometimes no,” and “usually, no,” and the final four items provided a 3-point scale (as per 4-point but with “not needed” removed). Community supportiveness was computed as the average of responses and converted to percentage scores. Items that caregivers indicated a specific barrier or support was not relevant to their child were excluded from the total percentage score. Child participation in community activities was computed as the percentage of activities in which the child participates, with higher scores indicating more activities. Child involvement or engagement in community activities was computed as the average of scores for responses, with higher scores indicating higher levels of engagement. The PEM-CY has demonstrated adequate internal consistency and test–retest reliability (Coster et al., 2011). Due to our small sample size and the volume of “not applicable” responses for this scale, we were unable to accurately determine reliability in our sample.

Vineland Adaptive Behavior Scales, Third Edition (VABS-III; Sparrow et al., 2016). The domain-level parent/caregiver form was administered to measure the adaptive level of functioning in children. The VABS-III domain-level form consists of five domains (communication, daily living skills, socialization, motor skills, and maladaptive behavior), each of which contains 40 items. Raw scores on subdomains of the scale are converted to percentile scores. An overall adaptive behavior composite score is calculated from the communication, daily living, and socialization items (*M* = 100, *SD* = 15). Scores of 70 or below reflect a low adaptive level, scores from 71 to 85 reflect moderately low, scores of 86–113 indicate adequate adaptive levels, scores of 115–129 reflect moderately high adaptive levels, and scores of 129 or more indicate a high adaptive level. The VABS-III has demonstrated high test–retest validity and acceptable levels of internal consistency for subdomains (Sparrow et al., 2016). Reliability in our sample was not established.

Parenting Stress Index, Fourth Edition (PSI-IV; Abidin, 2012). The PSI was administered to measure caregiver stress levels in relation to their child and to identify the domains in which these stress may originate from. The PSI asks caregivers to indicate their level of agreement to 101 items using a 4-point Likert scale to measure stressors across three key domains: child factors, caregiver factors, and situational or demographic factors. Scores were summed and percentiles calculated, with higher scores indicating higher stress in that domain. Scores above the 85th percentile on the PSI indicate a clinical level of stress, scores between 81 and 84 are considered high, and scores between 15 and 80 are considered typical levels of stress. The PSI has demonstrated good reliability (Abidin, 2012), with the total PSI score, child domain, and parenting domains demonstrating high reliability (Cronbach’s alpha = 0.94, 0.87, and 0.94, respectively), and subscales ranging from 0.63 (acceptability) to 0.89 (spouse/caregiving partner relationship). The life stress subscale did not have adequate reliability (Cronbach’s alpha = 0.36) and was not included in analyses.

Social Responsiveness Scale, Second Edition school-aged form (SRS-2; Constantino and Gruber, 2012). The SRS-2 was administered to quantify the severity of ASD symptom in children. This 65-item 4-point Likert scale measures the degree to which caregivers feel each item applied to their child in the preceding 6 months, measuring five areas: social awareness, social cognition, social communication, social motivation, and restricted interests and repetitive behavior. Items were summed, with higher scores indicating more severe deficiencies in symptom severity. Total scores of 76 or more indicate severe deficiencies, scores between 66 and 75 indicate moderate deficiencies, scores between 60 and 65 indicate mild deficiencies, and scores of 59 or less are considered to be within normal limits. The SRS-2 has demonstrated good construct validity and internal consistency with primary-school aged children (Wigham et al., 2012), with the total SRS score and the Social Communication Index demonstrating high reliability (Cronbach’s alpha = 0.94 and 0.94, respectively), and subscales ranging from 0.63 (awareness) to 0.88 (Communication) in our sample.

### Procedure

Ethical approval was provided by the Deakin Human Research Ethics Committee and the Victorian Department of Education and Training. Those who indicated interest in participating were provided with a plain language statement, and caregivers provided written informed consent while children gave verbal assent. Questionnaires were completed by caregivers while their child participated in testing sessions held at university campuses, football clubs, private clinics and school-based sessions across Victoria as part of the larger longitudinal project, or in some cases questionnaires were completed at home and returned by post.

### Statistical Analysis

All statistical analyses were conducted using IBM SPSS statistics version 25. Missing data of less than 5% were treated as per scoring instructions for PSI and, similarly, less than 10% missing data were treated as per scoring instructions for SRS-2. VABS-II does not allow any missing data, and PEM-CY is averaged to account for missing data. For data that exceeded the criterion for missing responses across participants, Little’s Missing Completely at Random test indicated that data were missing at random (*χ*^2^ = 307.46, *df* = 365, *p* = 0.99) and so list-wise deletion was used. Data were not normally distributed and the sample was small, so two-tailed Kendall’s tau correlations were conducted to identify significant relationships between variables. Hierarchical regression was conducted using variables significantly associated with child community involvement, child community attendance, and caregiver stress (isolation). Mahalanobis Distances were calculated and outliers with probability lower than 0.001 were removed. P-P plots were examined to assess whether residuals were normally distributed, and collinearity statistics (VIF and tolerance) and Durbin-Watson statistics were examined. All assumptions were met. Assuming the following parameters – large effect size, α error probability of 0.05, a maximum of six predictors and power of 0.80 – a total sample size of 46 was required.

## Results

### Participant Characteristics and Correlations

Four female and 44 male children aged 5–12 years (*M* = 8.41; *SD* = 2.16) with a full-scale IQ ranging from 41 to 134 (*M* = 87.8; *SD* = 20.83) participated. An additional eight participants did not complete the questionnaire items pertaining to the outcome measures, and so were not able to be included in the analyses. Independent t-tests and chi-square analyses did not identify significant differences between those with missing data on outcome measures. Caregivers consisted of 30 mothers and nine fathers, with nine caregivers not reporting their gender. Caregivers’ ages ranged from 30 to 52 years (*M* = 41.40, *SD* = 5.36). Fourteen caregivers (31%) scored above the 85th percentile on the PSI, indicating a clinical level of stress. All other participants were within the normal range. Means and standard deviations for study variables are found in Table 1, as are correlations between variables.

**Table 1:** Means, standard deviations, and correlations between study variables.

		Community environmental supportiveness (*n* = 48)	Child community attendance frequency (*n* = 48)	Child community involvement (*n* = 48)	Parenting stress isolation (*n* = 45)
Variable (*n*)	Mean (*SD*)	79.08 (11.07)	57.92 (16.24)	3.43 (0.84)	66 (24.93)
*Social responsiveness scale*					
Social awareness (45)	13.02 (3.31)	−0.21	−0.13	−0.22^*^	0.08
Social cognition (45)	19.00 (5.90)	−0.19	−0.16	−0.08	0.10
Social communication (45)	33.93 (10.10)	−0.29	−0.12	−0.19	0.21
Social motivation (45)	15.09 (5.53)	−0.15	−0.16	−0.21^*^	0.05
Restricted interests and repetitive behaviors (45)	20.33 (5.54)	−0.05	−0.14	−0.24^*^	0.13
Social communication and interaction (45)	81.04 (21.91)	−0.25	−0.11	−0.19	0.17
Total (45)	101.38 (26.26)	−0.22	−0.07	−0.19	0.18
*Parenting stress index*					
Distractibility (45)	85.44 (15.67)	−0.04	-	-	-
Adaptability (45)	85.93 (14.19)	−0.12	-	-	-
Reinforces caregiver (45)	62.09 (22.75)	−0.13	-	-	-
Demandingness (45)	85.33 (17.65)	−0.17	-	-	-
Mood (45)	79.49 (20.80)	−0.02	-	-	-
Acceptability (45)	82.22 (14.41)	−0.11	-	-	-
Child domain (45)	86.04 (12.45)	−0.15	-	-	-
Competence (45)	61.40 (25.12)	−0.09	-	-	-
Isolation (45)	66.00 (24.93)	−0.36^**^	-	-	-
Attachment (45)	47.89 (22.14)	0.02	-	-	-
Health (45)	74.29 (21.14)	−0.12	-	-	-
Role restriction (45)	70.89 (25.50)	−0.17	-	-	-
Depression (45)	65.36 (25.96)	−0.10	-	-	-
Spouse/caregiving partner relationship (45)	60.60 (27.50)	−0.10	-	-	-
Caregiving domain (45)	63.78 (22.93)	−0.14	-	-	-
Total (45)	77.29 (14.54)	−0.18	-	-	-
*Vinelands adaptive behavior scales*					
Adaptive behavior (47)	72.51 (9.60)	0.28	−0.14	0.31^**^	−0.33^**^
Communication (47)	75.11 (13.45)	0.18	−0.15	0.25^*^	−0.25^*^
Daily living (47)	74.43 (13.14)	0.32^*^	−0.11	0.31^**^	−0.25^*^
Socialization (47)	72.11 (10.11)	0.24	−0.11	0.22^*^	−0.25^*^
Motor skills (33)	82.76 (12.79)	0.27	−0.16	0.14	−0.09
Internalizing (46)	19.50 (2.24)	−0.07	−0.07	0.11	−0.01
Externalizing (46)	18.72 (2.65)	−0.33^*^	−0.03	0.09	0.16
Age of child (48)	8.41 (2.16)	−0.35^*^	−0.02	−0.10	0.02
FSIQ (44)	87.07 (20.48)	−0.04	−0.30^*^	0.01	0.10

Participant numbers varied across measures due to incomplete data.

* Significant at 0.05;

** Significant at 0.01.

All children with ASD had participated in some kind of neighborhood activity, however, 12% had never participated in a community event, 33% had never participated in organized physical activity, 2% had never participated in unstructured physical activity, 69% had never participated in classes or lessons outside of school, 88% had never participated in organizations, clubs, groups or leadership activities, 63% had never participated in religious activities, 22% had never “gotten together” with other children in the community, and 45% had never stayed overnight (i.e., for a sleepover, holiday, or camp).

Fifty-five percent of caregivers identified features of the environment as not supportive of their child’s community participation, and 27% of caregivers felt that information or equipment/supplies at community activities were not adequate for supporting their child’s participation. Social demands were identified most frequently as a barrier (35% of caregivers), followed by cognitive demands (33%), sensory demands (22%), physical demands (16%), relationships with peers, attitudes in the community and community safety (8%), and weather conditions (4%).

### Child Community Attendance

As shown in Table 1, the only significant correlation identified between child characteristics and community participation was child FSIQ (*p* = 0.004). To explore whether community supportiveness accounts for variations in child community attendance beyond the effects of child characteristics, a stepped multiple linear regression analysis was conducted with FSIQ entered at step 1 and community supportiveness in step 2. As shown in Table 2, child FSIQ significantly predicted child community attendance, *F*(1,42) = 6.18, *p* = 0.02, accounting for 13% of the variance in child community attendance. After controlling for child FSIQ, community supportiveness, entered in step 2, did not significantly predict child community attendance, *F*(2,41) = 3.25, *p* = 0.05, *F*-change = 0.40, *p* = 0.53.

**Table 2:** Predicting child community participation from child IQ and community supportiveness.

Variable	*B*	*β*	*p*	*sr*^2^	*R*	*R*^2^	*F*	*Sig.*
Step One					0.36	0.13	6.18	0.02
(constant)	5.11	-	<0.00	-				
FSIQ	−0.01	−0.36	0.02	−0.36				
Step Two					0.37	0.14	3.25	0.05
(constant)	4.53	-	<0.00	-				
FSIQ	−0.01	−0.35	0.02	−0.35				
Community supportiveness	0.01	0.09	0.53	0.10				

### Child Community Involvement

To explore whether community supportiveness accounts for variations in child community involvement beyond the effects of child characteristics, a stepped multiple linear regression analysis was conducted with social awareness, social motivation, restricted interests and repetitive behaviors and adaptive behavior entered at step 1, and community supportiveness in step 2. As shown in Table 3, child characteristics revealed a collective effect on child community involvement *F*(4,40) = 5.96, *p* = 0.001, which accounted for 37% of the variation in child community involvement. After controlling for child characteristics, community supportiveness, entered in step 2, further predicted child community involvement, *F*(5,39) = 6.21, *p* < 0.001, F-change = 0.4.90, *p* = 0.03. The combined predictors accounted for 44% of the variation in child community involvement. The individual predictors were examined further and indicated that adaptive behavior (*t* = 3.93, *p* < 0.001) and community supportiveness (*t* = 2.21, *p* = 0.03) were significant predictors in the model.

**Table 3:** Predicting child community involvement from child social responsiveness, adaptive behavior and restricted interests and repetitive behaviors.

Variable	*B*	*β*	*p*	*sr*^2^	*R*	*R*^2^	*F*	*Sig.*
Step One					0.61	0.37	5.96	0.001
(constant)	0.14	-	0.94	-				
Social awareness	0.02	0.24	0.17	0.22				
Social motivation	−0.01	−0.13	0.44	−0.12				
Restricted interests and repetitive behaviors	−0.02	−0.18	0.29	−0.17				
Adaptive behavior	0.05	0.56	<0.001	0.53				
Step Two					0.67	0.44	6.21	<0.001
(constant)	−1.21	-	0.51	-				
Social awareness	0.02	0.28	0.10	0.26				
Social motivation	−0.01	−0.11	0.50	−0.11				
Restricted interests and repetitive behaviors	−0.02	−0.22	0.19	−0.21				
Adaptive behavior	0.05	0.50	0.001	0.50				
Community supportiveness	0.02	0.28	0.03	0.33				

### Caregiver Stress

Caregiver perceptions of increased overall community support were associated with caregivers perceiving lower levels of isolation (*p* = 0.001). Correlation analyses, reported in Table 4, were run to identify child variables associated with caregiver isolation. Community supportiveness and child characteristics associated with caregiver isolation (child adaptive behavior) were added into a two-step hierarchical regression analysis with caregiver isolation as the criterion variable. The child predictor (adaptive behavior) was added in step 1, and community supportiveness was added in step 2. As shown in Table 5, the hierarchical regression model revealed that at step 1 there was a collective significant effect of adaptive behavior *F*(1,43) = 4.69, *p* = 0.04, with low adaptive behavior explaining 10% of the variance in caregiver isolation. Adding community supportiveness to the model resulted in a significant model *F*(2,42) = 8.01, *p* = 0.001. Community supportiveness explained 18% of the variance in caregiver isolation, and this change was significant (*p* = 0.003). The individual predictors were examined further and indicated that adaptive behavior was no longer a significant predictor when community supportiveness was included in the model (*t* = −1.45, *p* = 0.15), while community supportiveness was a significant predictor in the model (*t* = −3.21, *p* = 0.003).

**Table 4:** Correlations between caregiver isolation and child variables.

Variable (*n*)	Parenting stress isolation (*n* = 45)
*Social responsiveness scale*	
Social awareness (45)	0.08
Social cognition (45)	0.10
Social communication (45)	0.21
Social motivation (45)	0.05
Restricted interests and repetitive behaviors (45)	0.13
Social communication and interaction (45)	0.17
Total (45)	0.18
*Vinelands adaptive behavior scales*	
Adaptive behavior (47)	−0.33^**^
Communication (47)	−0.25^*^
Daily living (47)	−0.25^*^
Socialization (47)	−0.25^*^
Motor skills (33)	−0.09
Internalizing (46)	−0.01
Externalizing (46)	0.16
Age of child (48)	0.02
FSIQ (44)	0.10

* Significant at 0.05;

** Significant at 0.01.

**Table 5:** Predicting parenting isolation from child adaptive behavior and community supportiveness.

Variable	*B*	*β*	*p*	*sr*^2^	*R*	*R*^2^	*F*	*Sig.*
Step One					0.31	0.10	4.69	0.04
(constant)	128.67	-	0.00	-				
Adaptive behavior	−0.86	−0.31	0.04	−0.31				
Step Two					0.53	0.28	8.01	0.001
(constant)	184.57	-	0.00	-				
Adaptive behavior	−0.55	−0.20	0.15	−0.22				
Community supportiveness	−0.98	−0.44	0.003	−0.44				

## Discussion

The current study aimed to address gaps in the literature regarding community inclusion of children with ASD and their families through identifying correlates of child participation and involvement in community activities, caregiver perceptions of community supportiveness, and caregiver stress. Adaptive behavior and ASD symptom severity can impede the ability of children with ASD to meet the various cognitive, social, and communication demands of participation in community activities (Bar-Shalita et al., 2008; Hochhauser and Engel-Yeger, 2010; Obrusnikova and Cavalier, 2011; Poon, 2011; Shattuck et al., 2011; Thompson and Emira, 2011; Krieger et al., 2018; May et al., 2018). Our findings partially supported this former research by showing that community attendance and involvement rates of children with ASD were low and significantly associated with FSIQ (attendance) and adaptive behaviors and ASD symptom severity, such as social awareness, social motivation, and restricted interests and repetitive behaviors (involvement).

The relationships between child characteristics and child involvement were stronger than those with child attendance, indicating that child adaptive behaviors and ASD symptom severity may be more important for child engagement and involvement in community programs than for the attendance of children with ASD in community programs. Specifically, greater ASD symptom severity and fewer adaptive behaviors are linked to lower quality of community engagement but not lower quantity. The findings that FSIQ predicted child community attendance aligns with previous research finding the cognitive demands of community activities act as a barrier for children with ASD (Egilson et al., 2017); however, the lack of other significant predictors for child community attendance was unexpected. Previous research has shown a range of links for child attendance with child characteristics and community supportiveness (Egilson et al., 2017; Krieger et al., 2018), and the current findings conflict with this former literature.

In line with the social model of disability, our findings highlighted the role community supportiveness plays in child involvement in community activities. As expected, a high number of caregivers indicated community environments did not support their child’s participation. Regardless of a child’s symptom severity, community supportiveness significantly predicted child involvement, supporting the premise that children with ASD can experience successful involvement in their community with the right supports in place, or conversely, experience poor involvement when their community is not supportive. These findings fit with previous research indicating community factors, such as poor predictability, social and cognitive demands and low availability, and suitability of community services and staff (Egilson et al., 2017; Krieger et al., 2018), can disrupt child participation, and extend these findings through demonstrating that community supportiveness may be more important for involvement than the child characteristics themselves. These findings provide promising initial support that adapting community programs and activities for children with ASD may lead to increased involvement of these children. Of note, however, reduced child adaptive behavior significantly predicted child involvement irrespective of community supportiveness. This may indicate an area in which intensive supports and intervention may be needed to increase the ability of children with ASD to be fully involved in their community.

### Caregiver Stress

Caregiver stress in our sample was high, with one in three caregivers scoring above the clinical cut-off on the Parenting Stress Index. Previous research found caregivers who perceive social support received by themselves or their child as inadequate are more likely to experience high levels of stress (Siklos and Kerns, 2006; Siman-Tov and Kaniel, 2011), and our results suggest that caregivers of children with ASD who perceive their community as being unsupportive of their child’s participation in the community due to the presence of many barriers, low levels of helpfulness, and few resources available may experience higher levels of isolation. Similar to research of Hall (2012), in which child ASD symptom severity and community supportiveness accounted for 16% of the variance in family coping scores, community supportiveness explained 18% of the variance in caregiver isolation in the current study. Community supportiveness was not linked to other forms of stress in our sample. This may indicate that where other studies found other forms of low social supports (i.e., friends and family) were linked to high caregiver stress (Hall, 2012), community supportiveness may be less important for other forms of stress beyond feelings of isolation.

Of particular note is the finding that when variations in caregiver perceptions of community supportiveness were accounted for, child adaptive behaviors no longer significantly predict caregiver isolation. These findings suggest that low levels of community supports for child participation contribute more strongly to caregiver isolation than child functioning does, indicating that increasing community supportiveness of children with ASD may also decrease caregiver isolation. This fits with previous qualitative research in which caregivers indicated low levels of community support resulted in caregivers taking on an advocacy role for their child, which left them feeling labeled and isolated (Resch et al., 2010).

### Limitations

The research findings need to be interpreted in the context of a number of limitations. First, given the current study was limited to cross-sectional data, directionality cannot be confirmed. In particular, it is unclear at this stage whether caregivers who feel isolated are more likely to perceive increased barriers to their child’s participation, or whether barriers to child participation lead to feelings of isolation in caregivers. It is also possible that this relationship is reciprocal, with increased barriers leading to feelings of isolation, and increased feelings of isolation leading to an increasingly negative view of community supportiveness for child inclusion; however, further research is needed to establish directionality. Second, Wechsler tests of Intelligence can underestimate cognitive functioning. This study’s aim was to control variations in FSIQ and, therefore, this underestimation is unlikely to impact results; however, future research could consider examining other non-verbal measures of cognitive functioning in relation to community participation. Finally, the sample size for this study was small and, therefore, only powered to detect large effects, consisted of caregivers of predominantly male children, and half of the sample consisted of children with autism recruited from a community football program. Further, reliability of PEM-CY and Vinelands was not established for our sample, and variations in reliability have been noted for the PEM-CY previously (see Coster et al., 2011; Simpson et al., 2019). Replication in a larger and more diverse population would address these limitations.

## Conclusion

In summary, these findings suggest that lower perceived levels of community supportiveness may reduce the involvement of children with ASD in community activities and increase feelings of isolation in their caregivers. Specifically, children with ASD may experience increased inclusion in programs that cater for varying communication, cognitive and social abilities, and in addressing key barriers to participation of children, caregivers may experience reduced feelings of isolation. Disruptions to adaptive behaviors in children with ASD may pose particular challenges for children with ASD, and further research exploring intensive intervention and supports is warranted.

The findings that child characteristics and community supportiveness may have more impact on child involvement or engagement in community activities than on attendance raise questions as to whether reduced quality of engagement in community activities in children with ASD disrupts the benefits of regular participation. Future research delineating participation and involvement could explore if low engagement or involvement in community activities results in lower levels of beneficial outcomes in children with ASD despite regular participation, and test whether specific program attributes related to accessibility and inclusivity impact child involvement. Further research is also needed to identify and evaluate the effective modifications to community programs in these key areas and to measure their collective impact on caregiver perceptions of community supportiveness and caregiver isolation. Community sports that are tailored to facilitate the inclusion of children with ASD have been increasingly recognized over the past decade as a promising intervention medium (Rinehart et al., 2018; Howells et al., 2019). The findings that adaptive behavior and community supportiveness support child involvement in community settings play an important role in providing an evidence-based approach for inclusion in community sports settings, while seeking to ensure that the benefits of these approaches extend beyond merely boosting attendance of a community program, and instead reflect a deeper engagement and connection within these settings. This study provides clear insights into the potential for inclusive and adapted community programs to facilitate active engagement and participation in children with ASD, while reducing isolation stress in caregivers.

## Data Availability Statement

The raw data supporting the conclusions of this article will be made available by the authors, without undue reservation.

## Ethics Statement

The studies involving human participants were reviewed and approved by Deakin University Human Research Ethics Committee. Written informed consent to participate in this study was provided by the participants’ legal guardian/next of kin.

## Author Contributions

All authors were involved in data interpretation and manuscript drafting. All authors read and approved the final manuscript.

### Conflict of Interest

All financial, commercial, or other relationships that might be perceived by the academic community as representing a potential conflict of interest must be disclosed. If no such relationship exists, authors will be asked to confirm the following statement: NR, JM, and the Deakin Child Study Centre currently receive funding from the Moose Foundation, Victorian Department of Education and Training, MECCA Brands, Wenig Family, Geelong Community Foundation, and Grace & Emilio Foundation to conduct research in the field of neurodevelopmental disorders and inclusion. NR also receives funding from the Ferrero Group Australia as part of its Kinder + Sport pillar of Corporate Social Responsibility initiatives to promote active lifestyles among young people. NR has previously received donations from Vic Health and Bus Association Victoria received previous speaker honorarium from Novartis (2002), Pfizer (2006), and Nutricia (2007), and is a Director of the Amaze Board (Autism Victoria). None of the companies or organizational bodies listed above had a role in this research including the collection, analysis, and interpretation of data, in writing of the manuscript, and/or in the decision to submit the article for publication. BD, CS, and EL are research fellows in the Deakin Child Study Centre.

The remaining authors declare that the research was conducted in the absence of any commercial or financial relationships that could be construed as a potential conflict of interest.
